# Mass spectrometric identification of immunogenic SARS-CoV-2 epitopes and cognate TCRs

**DOI:** 10.1073/pnas.2111815118

**Published:** 2021-11-01

**Authors:** Ke Pan, Yulun Chiu, Eric Huang, Michelle Chen, Junmei Wang, Ivy Lai, Shailbala Singh, Rebecca M. Shaw, Michael J. MacCoss, Cassian Yee

**Affiliations:** ^a^Department of Melanoma Medical Oncology, The University of Texas MD Anderson Cancer Center, Houston, TX 77054;; ^b^Department of Genome Sciences, University of Washington, Seattle, WA 98195;; ^c^Department of Biologics Development, The University of Texas MD Anderson Cancer Center, Houston, TX 77054;; ^d^Department of Immunology, The University of Texas MD Anderson Cancer Center, Houston, TX 77054

**Keywords:** SARS-CoV-2, MHC peptide, mass spectrometry, T cells, TCR-T

## Abstract

Severe acute respiratory syndrome coronavirus 2 (SARS-CoV-2) infections elicit both humoral and cellular immune responses. For the prevention and treatment of COVID-19, the disease caused by SARS-CoV-2, it has become increasingly apparent that T cell responses are equally if not more important than humoral responses in mediating recovery and immune protection. One major challenge in developing T cell–based therapies for infectious and malignant diseases has been the identification of immunogenic epitopes that can elicit a meaningful T cell response. Traditionally, this has been achieved using sophisticated in silico methods to predict putative epitopes deduced from binding affinities. Our studies find that, in contrast to current convention, “immunodominant” SARS-CoV-2 peptides defined by such in silico methods often fail to elicit T cell responses recognizing naturally presented SARS-CoV-2 epitopes. We postulated that immunogenic epitopes for SARS-CoV-2 are best defined empirically by directly analyzing peptides eluted from the naturally processed peptide–major histocompatibility complex (MHC) and then validating immunogenicity by determining whether such peptides can elicit T cells recognizing SARS-CoV-2 antigen-expressing cells. Using a tandem mass spectrometry approach, we identified epitopes derived from not only structural but also nonstructural genes in regions highly conserved among SARS-CoV-2 strains, including recently recognized variants. Finally, there are no reported T cell receptor–engineered T cell technology that can redirect T cell specificity to recognize and kill SARS-CoV-2 target cells. We report here several SARS-CoV-2 epitopes defined by mass spectrometric analysis of MHC-eluted peptides, provide empiric evidence for their immunogenicity, and demonstrate engineered TCR-redirected killing.

## Significance

Durable protection against COVID-19 infection may be achieved by generating robust T cell responses to severe acute respiratory syndrome coronavirus 2 (SARS-CoV-2) and emerging SARS-CoV-2 variants; for those infected, effective treatments are urgently needed. For these strategies to be successful, accurate identification of T cell epitopes is critical. In this study, we used major histocompatibility complex immune precipitation, acid elution, and tandem mass spectrometry to define the SARS-CoV-2 immunopeptidome for membrane glycoprotein (MGP) and the nonstructural protein. Furthermore, taking advantage of a highly robust endogenous T cell workflow, we verify the immunogenicity of these MS-defined peptides by in vitro generation of MGP and NSP13 peptide-specific T cells and confirm T cell recognition of MGP or NSP13 endogenously expressing cell lines.

Severe acute respiratory syndrome coronavirus 2 (SARS-CoV-2), the highly transmissible respiratory virus responsible for the COVID-19 pandemic outbreak, continues to render significant, lasting impact on global public health and has created an urgent need to develop accurate immunodiagnostics, and effective treatment strategies (1, 2). Rapid dissemination of the SARS-CoV-2 genomic sequence first revealed by Zhang Yongzhen led to large-scale efforts around the world to develop a protective vaccine that could elicit humoral (antibody) and cellular (T cell) responses (3). It follows that the identification of immunogenic epitopes of SARS-CoV-2 recognized by the human immune system would be critical for rational vaccine development.

Using in silico prediction algorithms, several investigators have amassed extensive panels of class I– and class II–restricted epitopes to probe SARS-CoV-2–specific T cell responses, in some cases, combining these with overlapping “megapools” spanning conserved regions of the genome (4, 5). These peptides have been used to track responses in infected and convalescent individuals (6, 7), to design multiepitope vaccines, and, directly or indirectly, to measure the breadth and severity of COVID-19 disease (7–13). While these studies have uncovered insights into the T cell immunobiology of COVID-19, the accuracy of T cell responses using in silico predicted responses and overlapping long peptide pools is diminished by a failure to consider whether such epitopes are immunogenic. An immunogenic epitope in this sense is defined as a peptide that is known to be presented by self–major histocompatibility complex (MHC), and is capable of eliciting T cells of sufficient affinity that such T cells can recognize target cells endogenously expressing antigen and presenting the antigen-derived peptide in the context of an MHC with sufficient surface density as to sensitize the target cell to peptide-specific T cell–mediated recognition. In essence, an immunogenic epitope of SARS-CoV-2 requires direct sequencing of peptides presented by MHC as well as empiric validation of T cell immunogenicity.

This study uses tandem mass spectrometry (MS) to identify T cell epitopes of SARS-CoV-2 following peptide elution from the MHCs of SARS-CoV-2–expressing cells, and empirically validates immunogenicity by in vitro generation of SARS-CoV-2–specific cytotoxic T lymphocytes (CTLs). Enabling technology developed by our group for the isolation of rare tumor-reactive T cells from very low precursor frequency populations in the peripheral blood was applied (14). We present data on the identification of five immunogenic epitopes of a highly conserved region of membrane glycoprotein (MGP) and the nonstructural protein region of the SARS-CoV-2 genome and demonstrate that such MGP-65–specific and NSP13-specific CTLs recognize and kill SARS-CoV-2 antigen-expressing target cells; we further sequence the T cell receptor (TCR) alpha and beta chains and demonstrate that specificity can be transferred by engineering expression of this TCR in polyclonal lymphocytes.

## Results

### SARS-CoV-2 Peptides Defined “In Silico” Fail to Elicit T Cells That Recognize SARS-CoV-2 Antigen-Expressing Targets.

**Fig. 1.**
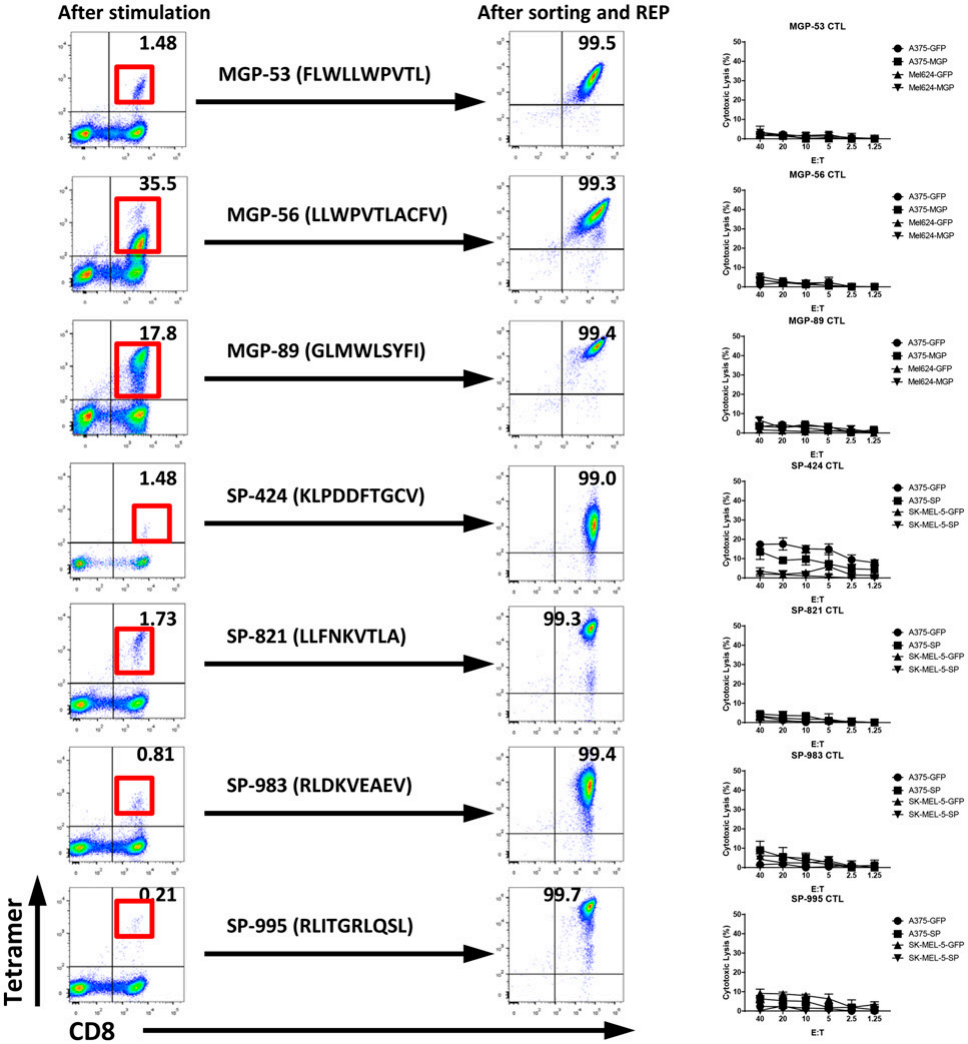
T cell generation and functional validation for predicted SARS-CoV-2 HLA-A0201–restricted peptide. Three predicted MGP HLA-A0201 peptides, MGP-53 (FLWLLWPVTL), MGP-56 (LLWPVTLACFV), and MGP-89 (GLMWLSYFI), and four predicted spike protein (SP) HLA-A0201 peptides, SP-424 (KLPDDFTGCV), SP-821 (LLFNKVTLA), SP-983 (RLDKVEAEV), and SP-995 (RLITGRLQSL), were selected for antigen-specific T cell generation using the ETC generation workflow. The peptide-pulsed mature DCs were cocultured with autologous PBMCs from HLA-A0201+ healthy donors. After two rounds of stimulation, CD8+ and Tetramer+ T cells were induced (*Left*). After sorting and a rapid expansion protocol (REP) for CD8+ and Tetramer+ T cells for 2 wk, high-purity specific CTLs were expanded (*Middle*). The antigen-specific cytolysis was analyzed with standard CRA using MGP, SP, or GFP force expressing HLA-A0201 cell lines (A375-MGP, A375-SP, A375-GFP, Mel624-MGP, Mel624-GFP, SK-MEL-5-SP, and SK-MEL-5-GFP) as targets (*Right*).

As an initial screen of known predicted epitopes for immunogenicity, we selected class I–restricted peptides to the SARS-CoV-2 Spike protein and MGP based on a literature search of studies where such “in silico” predicted peptides were described as “immunodominant.” These peptides have previously been reported to be “immunodominant” on the basis of their ability to generate high levels of peptide-specific responses from the peripheral blood mononuclear cells (PBMCs) of COVID-19+ patients and, surprisingly, in some healthy donors as well (apparently as a result of cross-reactive responses from T cells elicited in the past to nonpathogenic SARS viruses) (4, 7, 15–22). We synthesized four of these spike protein peptides and three of the MGP peptides. Using the endogenous T cell (ETC) generation workflow (see *Materials and Methods*), we generated individual T cell cultures against all four Spike peptides and all three MGP peptides (Fig. 1). However, when these highly enriched (>80% Tetramer+) T cell cultures were tested against HLA-matched target cells engineered to express the relevant SARS-CoV-2 Spike protein or MGP, no evidence of target cell killing was observed (Fig. 1). We postulated that these in silico predicted peptides were not endogenously presented, and that a more accurate means of identifying endogenously presented, immunogenic epitopes would be desirable and could be achieved by directly eluting and sequencing peptides from the MHC of SARS-CoV-2–expressing cells.

### Profiling of the MHC Class I–Restricted Epitope of SARS-CoV-2.

The antigen discovery platform for SARS-CoV-2 comprises four steps: 1) peptide elution and identification with MS for SARS-CoV-2 targets, 2) ETC generation workflow to elicit peptide-specific CTLs, 3) empiric validation of antigen-specific CTLs against SARS-CoV-2 targets, and 4) SARS-CoV-2–specific TCR engineered T cell (TCR-T) development (*SI Appendix*, Fig. S1). In order to elute and sequence the MHC-bound peptide derived from SARS-CoV-2, the SARS-CoV-2 genes were overexpressed in targets cells with different HLA allele expressions. Lentiviral expression vectors spanning highly conserved regions of SARS-CoV-2 regions—MGP or nonstructure protein helicase (NSP13) (10, 23)—were constructed and used to infect target cell lines A375 (HLA-A0101/0201), Mel624 (HLA-A0201), RPMI-7951 (HLA-A0101/0201), Hs-578T (HLA-A0301/A2402), and M14 (HLA-A1101/2402). Following puromycin selection and expansion of MGP or NSP13 stably expressing cell lines, purity of over 80% was achieved (*SI Appendix*, Fig. S2). The MGP- or NSP13-expressing cell lines were expanded to 300 million to 500 million, harvested, lysed with Nonidet P-40 detergent lysis buffer, and subjected to total HLA class I immunoprecipitation (anti–HLA-A, B, C) and acid elution, followed by tandem MS to analyze the HLA-bound peptides.

**Fig. 2.**
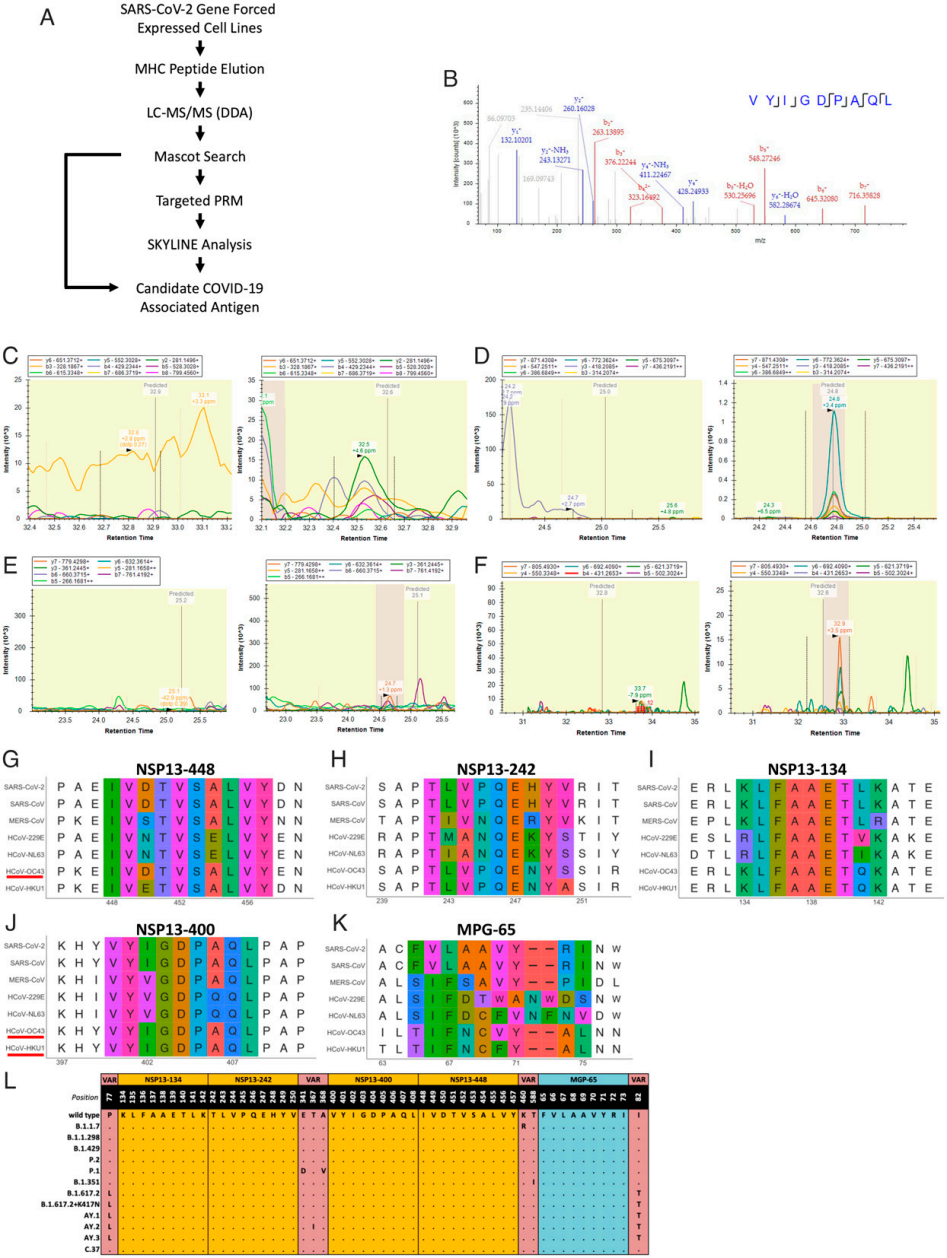
SARS-CoV-2–derived HLA class I peptide identification with MS. (*A*) Schematic representation of HLA-I peptide identification. MS/MS spectra of immunopeptidome and proteome analysis were searched against the Swiss-Prot human and virus protein database and filtered at a 1% false discovery rate. (*B*) MS/MS annotation for NSP13-400 peptide (VYIGDPAQL). (*C–F*) PRM analysis for NSP13-448 peptide (IVDTVSALVY), NSP13-242 peptide (TLVPQEHYV), NSP13-134 peptide (KLFAAETLK), and MGP-65 peptide (FVLAAVYRI). MS1 XIC areas and MS/MS for each targeted peptide were plotted using Skyline software. (*Left*) Negative control. (*Right*) Sample. (*G–K*) The multiple sequence alignment of the four candidate peptide sequences to all coronaviruses known to infect humans. (*L*) Four mutations were reported from NSP13 protein: E341D, A368V (P.1 lineage), K460R (B.1.1.7 lineage), T588I (B.1.351 lineage), and T367I (AY.2). Five mutations were reported from MGP protein: I82T (B.1.617.2, B.1.617.2+K417N, AY.1, AY.2, and AY.3). VAR, variants; a dot (.) indicates the same amino acid in that position.

We initially analyzed the eluted HLA-bound peptides derived from the SARS-CoV-2 targets established above by using data-dependent analysis liquid chromatography (LC) tandem MS (DDA MS/MS). The eluted spectra were searched using the Mascot search engine node (version 2.6) within the Proteome Discoverer (version 2.3) processing workflow with the Swiss-Prot human proteome database (version 2020_05) followed by the virus proteome database (version 2020_05). To reduce false positive hits from human proteome, the “Spectrum Confidence Filter” node within the Proteome Discoverer processing workflow filtered out all spectra with highly confident peptide-spectrum matches annotated from the human proteome. The remaining spectra were further searched against the virus proteome (Fig. 2*A*). In total, 12,770 MS/MS were acquired, and 9,731 peptide-spectrum matches were annotated, yielding 357 peptides with Mascot ions scores ≥ 25. Among the 357 peptides, a peptide derived from NSP13 (NSP13-400, VYIGDPAQL) with a Mascot ions score = 27 (Fig. 2*B*) was annotated. This peptide was eluted from M14-NSP13 cells (HLA-A1101/A2402). From the HLA binding prediction using the Immune Epitope Database tool, the NSP13-400 peptide scored high predicted binding affinity to the HLA-A2402 allele (Table 1), suggesting that NSP13-400 peptide is likely to be presented by HLA-A2402.

**Table 1.** Summary of identified SARS-CoV-2 epitopes

Eluted peptide	Protein ID	MS method	HLA allele	Binding prediction (nM)	Consensus region
VYIGDPAQL	NSP13-400	DDA	A2402	325.8	Yes
KLFAAETLK	NSP13-134	PRM	A0301	9.9	Yes
TLVPQEHYV	NSP13-242	PRM	A0201	72.2	Yes
IVDTVSALVY	NSP13-448	PRM	A0101	356.9	Yes
FVLAAVYRI	MGP-65	PRM	A0201	38.1	Yes

To enable more comprehensive profiling of potential HLA class I–restricted peptides from SARS-CoV-2, we further analyzed eluted peptides by parallel reaction monitoring MS (PRM-MS) to focus on predicted high-probability HLA-binding peptides derived from SARS-CoV-2 but not successfully detected by the DDA approach. Prior to the PRM-MS, 10 predicted high-potential HLA-A0101–, HLA-0201–, or HLA-A0301–binding peptides from MGP or NSP13 were selected (*SI Appendix*, Table S1). For the eluted peptide from target cell lines, precursor ion inclusion lists of 10 potential peptides were generated using Skyline, and we targeted and monitored these 10 peptides using nanoflow LC-PRM-MS with high mass accuracy and resolution. Pierce Peptide Retention Time Calibration Mixture peptides were used to monitor retention time drifts and adjust the scheduled PRM method. We first generated a spectral library using synthetic peptides, and we used both synthetic peptides and Pierce Peptide Retention Time Calibration Mixture peptides to define iRT, a normalized dimensionless peptide-specific value, to accurately predict retention time of each targeted peptide. We detected IVDTVSALVY (NSP13-448) with Dot-product (Dotp) = 0.58 and an average product ion ppm error of +4.6 ppm at predicted time 32.6 min (Fig. 2 *C*, *Right*) in target cell line RPMI-7951-NSP13, and no peak was detected in the negative control cell line RPMI-7951-GFP at predicted time 32.9 min (Fig. 2 *C*, *Left*). TLVPQEHYV (NSP13-242) with Dotp = 0.76 and an average product ion ppm error of +3.4 ppm at predicted time 24.8 min (Fig. 2 *D*, *Right*) was detected at target cell line A375-NSP13, and no peak was detected in the negative control cell line A375-GFP at predicted time 25.9 min (Fig. 2 *D*, *Left*). KLFAAETLK (NSP13-134) with Dotp = 0.67 and an average product ion ppm error of +1 ppm at predicted time 25.1 min was detected in target cell line Hs-578T-NSP13 (Fig. 2 *E*, *Right*), and no peak was detected in the negative control cell line Hs-578T-GFP at predicted time 25.2 min (Fig. 2 *E*, *Left*). The same rules applied. From A375-MGP, we detected FVLAAVYRI (MGP-65) with Dotp = 0.91 and an average product ion ppm error of +2.5 ppm at the predicted time 32.7 min (Fig. 2 *F*, *Right*), and no peak was detected in the negative control cell line A375-GFP at predicted time 32.8 min (Fig. 2 *F*, *Left*). These XIC MS2 analyses reported sufficient well-defined peaks in the positive control and no peaks showing in the negative control (matrix blank), suggesting that these targeted peptides exist in eluted peptide samples.

To evaluate whether these five candidate SARS-CoV-2 HLA class I–restricted peptides identified with DDA or PRM-MS are homologous to other coronaviruses including SARS-CoV, Middle East respiratory syndrome coronavirus (MERS-CoV), and four other coronaviruses, 229E, NL63, OC43, and HKU1, multiple sequence alignment analysis was performed. NSP13-242 (Fig. 2*H*), NSP13-134 (Fig. 2*I*), and MGP-65 (Fig. 2*K*) show a high degree of homology to the sequence of SARS-CoV. NSP13-400 (Fig. 2*J*) shows a high degree of homology to the sequence of SARS-CoV, HCoV-OC43 and HCoV-HKU1 (underlined in red). NSP13-448 (Fig. 2*G*) shows a high degree of homology to the sequence of SARS-CoV, HCoV-OC43 (underlined in red). In order to evaluate whether these five candidates are homologous to noncoronavirus species, the peptides were analyzed by using Basic Local Alignment Search Tool searches to identify all potential source proteins. The top 250 hits for each target sequence reported up to 88.99% identity or 100% identity but coverage up to 88.99% or 100% identity to related coronavirus, indicating these five candidate peptides are only homologous with coronavirus but no other species (Datasets S1–S5).

### Newly Defined Epitopes Are Found in Highly Conserved Regions of SARS-CoV-2 and SARS-CoV-2 Variants.

Olvera et al. (24) recently described the development of a COVID-19 vaccine using the overlapping of SARS-CoV-2 consensus sequences. Olvera et al. utilized an entropy-based calculation on more than 1,700 viral genome entries in the National Center for Biotechnology Information database and encompassed all described SARS-CoV-2 open reading frames (ORFs), including recently described frame-shifted and length-variant ORFs. The Nextstrain project (https://nextstrain.org), an open-source project that provides a continually updated view of publicly available data alongside powerful analytic and visualization tools to aid epidemiological understanding and improve outbreak response, provides a means to analyze genetic diversity across the SARS-CoV-2 genome. Using both of these sources, we verified that these five peptides were located in a highly conserved region of the SARS-CoV-2 genome (*SI Appendix*, Fig. S3). Recently, genetic variants of SARS-CoV-2 have emerged on a global scale, for example, mutation 23403A>G-(D614G) located on the spike protein, believed to render SARS-CoV-2 more infectious (25, 26). To investigate whether recently reported variants are located within these five candidate SARS-CoV-2 HLA class I–restricted epitopes, we focused on variants of concern first described in the United Kingdom (B.1.1.7), Denmark (B.1.1.298), United States (B.1.429), Brazil and Japan (P.2 and P.1), and South Africa (B.1.351) and on the Delta (B.1.617.2, B.1.617.2+K417N, AY.1, AY.2, AY.3) and Lambda (C.37) variants. For the NSP-13 region, there are several mutations, E341Y and A368V, present in P.1 variants; one mutation, T367I, present in AY.2; one mutation, K460R, present in the B.1.1.7 variant; and one mutation, T588I, present in the B.1.351 variant; none of these mutations overlap with these four NSP-13 HLA class I–restricted peptides. I82T is a mutation present in Delta variants (B.1.617.2, B.1.617.2+K417N, AY.1, AY.2, and AY.3) and found within the MGP region but does not overlap with the MGP-65 peptide identified in this study(Fig. 2*L*). Hence, MGP-65 is expected to remain effective even among Delta variants. This finding supports the observation that the identified HLA class I peptides derived from MGP or NSP13 are highly conserved among current and newly emergent variants.

### MGP-65 Peptide-Specific Cytotoxic T Cells Generated from the Peripheral Blood Recognize SARS-CoV-2 MGP-Expressing Target Cells.

**Fig. 3.**
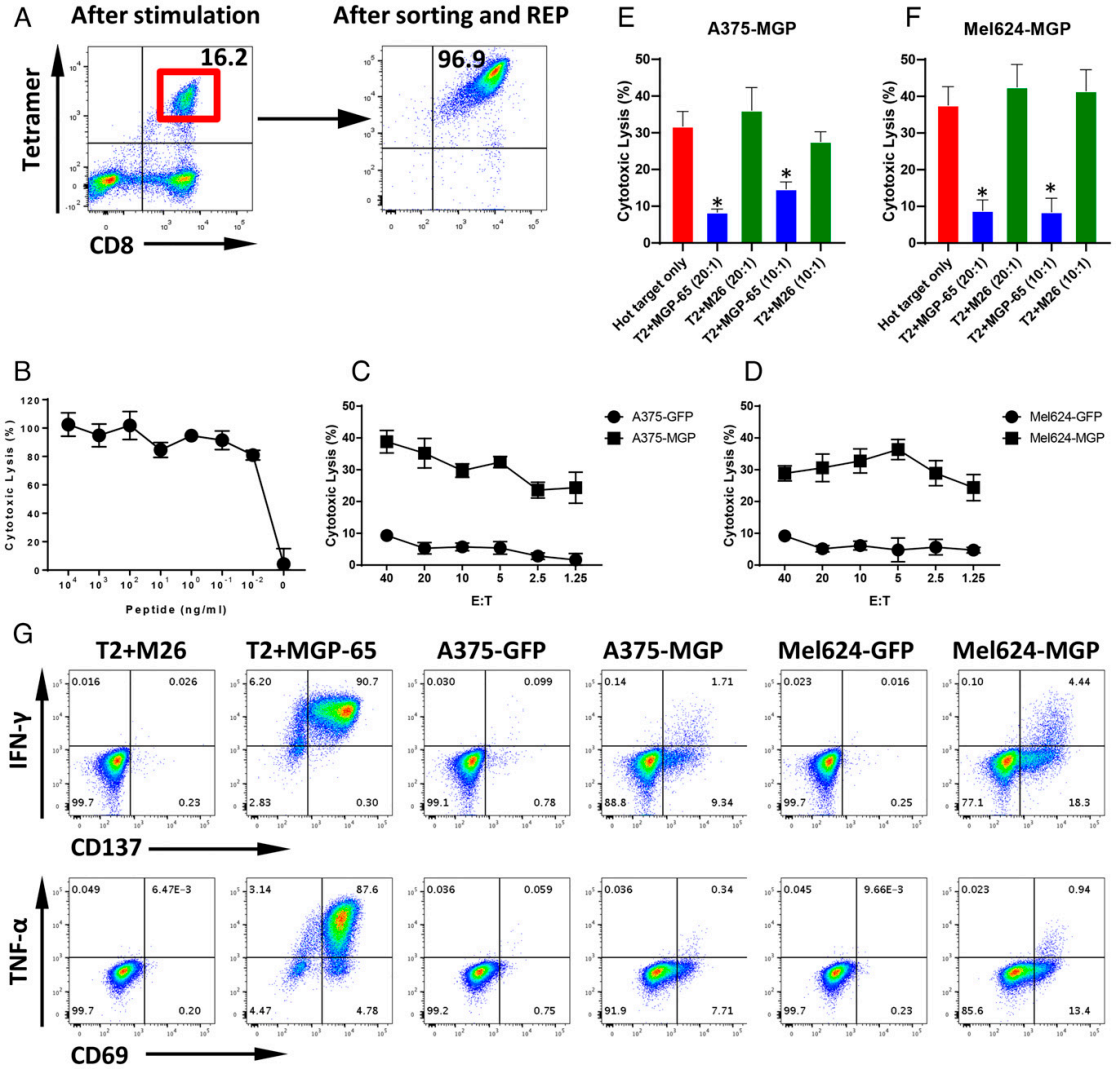
T cell generation and functional validation of MGP-derived HLA-A0201 peptide MGP-65. (*A*) Mature DCs derived from an HLA-A0201 healthy donor were pulsed with MGP-65 peptide (FVLAAVYRI) and cocultured with autologous PBMCs. After two rounds of stimulation, small CD8+ and MGP-65 Tetramer+ populations were observed (*Left*). CD8+ and MGP-65 Tetramer+ cells were then sorted and expanded using a standard REP. After expansion for 2 wk, high-purity CTLs (Tetramer+ population over 90%) were generated (*Right*). (*B*) The ^51^Cr-labeled T2 cells pulsed with various concentrations of MGP-65 peptide were cocultured with MGP-65–specific CTLs at a 20:1 E:T ratio. The lysis ability of MGP-65–specific CTLs was detected with standard CRA. The data are shown as average of triplicate. (*C* and *D*) The ^51^Cr-labeled MGP or GFP force expressing HLA-A0201 cell lines A375 (A375-MGP and A375-GFP) and Mel624 (Mel624-MGP and Mel624-GFP) were cocultured with MGP-65–specific CTLs at various E:T ratios (from 40:1 to 1.25:1). The lysis ability of MGP-65–specific CTLs to different targets was detected with standard CRA. The data are shown as average of triplicate. (*E* and *F*) Cold target inhibition assay. The ^51^Cr-labeled A375-MGP and Mel624-MGP were used as hot targets. Nonradiolabeled T2 cells pulsed with MGP-65 peptide or M26 irrelevant peptide were used as cold targets. The C:H ratio was 10:1 or 20:1. MGP-65–specific CTLs were cocultured with hot targets alone or hot targets together with cold targets at a 20:1 E:hot T ratio. The lysis ability of MGP-65–specific CTLs was detected with standard CRA. The data are shown as average of triplicate. (*G*) ICS assay. MGP-65–specific CTLs were cocultured with T2 pulsed with MGP-65 peptide or M26 irrelevant peptide, as well as A375-MGP, A375-GFP, Mel624-MGP, and Mel624-GFP at a 10:1 E:T ratio in the presence of Brefeldin A (BFA) overnight. After incubation, the levels of IFN-γ and TNF-α, as well as TCR pathway downstream activated marker CD137 and CD69, were detected using flow cytometry.

Following leukapheresis, HLA-A0201 healthy donor PBMCs were stimulated with MGP-65 peptide (FVLAAVYRI)–pulsed autologous dendritic cells (DCs). After two rounds of stimulation, MGP-65-A2 Tetramer+ staining populations were detected (Fig. 3*A*). About 33 wells from one 48-well plate showed clear MGP-65 peptide Tetramer+ CD8+ T cell populations (*SI Appendix*, Fig. S4), indicating that MGP-65 peptide-specific T cells are easily expanded with cognate peptide stimulation, even in the PBMCs of healthy donors without a history of SARS-CoV-2 infection. Expanded MGP-65 CTLs were tested functionally using standard ^51^Cr release assays (CRAs). T2 cells (HLA-A0201), pulsed with titrated amounts of MGP-65 peptide, elicited CTL recognition and killing at peptide concentrations as low as 10 pM (Fig. 3*B*), indicating very high recognition affinity of MGP-65 CTLs for cognate peptide.

To verify that MGP-65–specific CTLs recognized the endogenously presented cognate peptide, HLA-A0201+ target cells were engineered to express the SARS-CoV-2 MGP gene (A375-MGP, Mel624-MGP). MGP-65–specific CTLs were able to lyse A375-MGP and Mel624-MGP cell lines, but not A375-GFP and Mel624-GFP control cell lines (Fig. 3 *C* and *D*). To further confirm that target recognition of MGP-65–specific CTLs was through engagement of endogenously presented cognate peptide, a cold target inhibition assay was performed. The ^51^Cr-pulsed MGP-expressing cells were radiolabeled with ^51^Cr, while MGP-65– or M26 irrelevant peptide-pulsed T2 cells were left unlabeled and used as cold targets or control cold targets, respectively. When adding cold targets at both 20:1 and 10:1 cold to hot target (C:H) ratios, the cytotoxicity of MGP-65–specific CTLs for radiolabeled MGP-65 peptide targets was significantly inhibited (Fig. 3 *E* and *F*). However, there was no inhibition if control cold targets were added, indicating MGP-65 CTLs were able to then lyse MGP-65 targets via recognition of endogenously presented cognate peptide. These data provided further evidence that MGP-65 peptide is the natural endogenously presented MHC peptide.

To further evaluate function of the MGP-65–specific CTLs, the intracellular staining (ICS) assay was performed to detect IFN-γ and TNF-α production. Coculture of MGP-65–specific CTLs with MGP-65 peptide-pulsed or MGP-engineered target cells demonstrated specific recognition with significantly enhanced production of IFN‐γ and TNF‐α compared with control targets pulsed with irrelevant peptide or engineered to express control GFP (Fig. 3*G*). A commensurate increase in T cell activation markers, CD137 and CD69, was also specifically and significantly elevated compared with the control group (Fig. 3*G*), when encountering relevant SARS-CoV-2 targets.

### NSP13-242 Peptide-Specific Cytotoxic T Cells Generated from the Peripheral Blood Recognize SARS-CoV-2 NSP13-Expressing Target Cells.

**Fig. 4.**
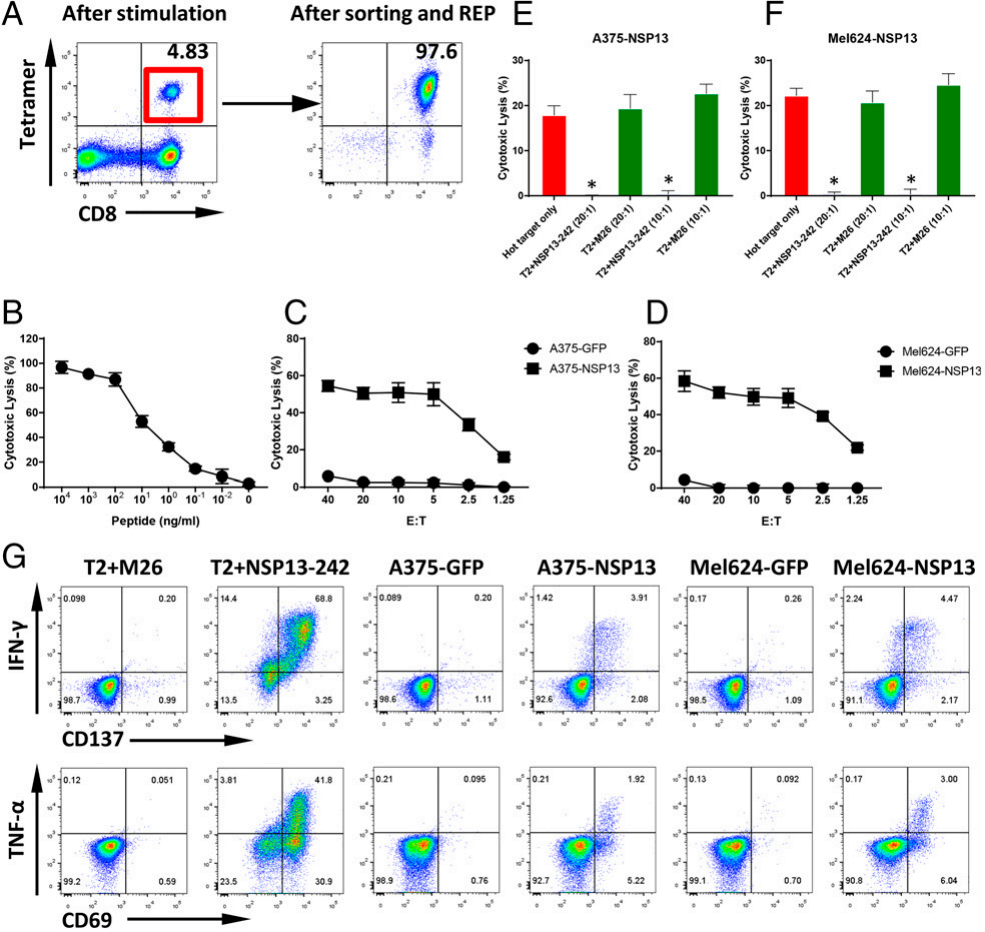
T cell generation and functional validation of nonstructure protein NSP13-derived HLA-A0201 peptide NSP13-242. (*A*) PBMCs from an HLA-A0201 healthy donor were cocultured with NSP13-242 peptide (TLVPQEHYV)–pulsed autologous DCs. After two rounds of stimulation, CD8+ and NSP13-242 Tetramer+ T cells were induced (*Left*). CD8+ and Tetramer+ T cells were sorted and then expanded with REP for 2 wk to generate high-purity NSP13-242–specific CTLs (*Right*). (*B*) The ^51^Cr-labeled T2 cells pulsed with various concentrations of NSP13-242 peptide were cocultured with NSP13-242–specific CTLs at a 20:1 E:T ratio. The lysis ability of NSP13-242–specific CTLs was detected with standard CRA. The data are shown as average of triplicate. (*C* and *D*) The ^51^Cr-labeled NSP13 or GFP force expressing HLA-A0201 cell lines A375 (A375-NSP13 and A375-GFP) and Mel624 (Mel624-NSP13 and Mel624-GFP) were cocultured with NSP13-242–specific CTLs at various E:T ratios (from 40:1 to 1.25:1). The lysis ability of NSP13-242–specific CTLs to different targets was detected with standard CRA. The data are shown as average of triplicate. (*E* and *F*) Cold target inhibition assay. The ^51^Cr-labeled A375-NSP13 and Mel624-NSP13 were used as hot targets. Nonradiolabeled T2 cells pulsed with NSP13-242 peptide or M26 irrelevant peptide were used as cold targets. The C:H ratio was 10:1 or 20:1. NSP13-242–specific CTLs were cocultured with hot targets alone or hot targets together with cold targets at a 20:1 E:hot T ratio. The lysis ability of NSP13-242–specific CTLs was detected with standard CRA. The data are shown as average of triplicate. (*G*) ICS assay. NSP13-242–specific CTLs were cocultured with T2 pulsed with NSP13-242 peptide or M26 irrelevant peptide, as well as A375-NSP13, A375-GFP, Mel624-NSP13, and Mel624-GFP at a 10:1 E:T ratio in the presence of BFA overnight. After incubation, the levels of IFN-γ and TNF-α, as well as TCR pathway downstream activated marker CD137 and CD69, were detected using flow cytometry.

In contrast to structural proteins such as MGP and Spike protein, nonstructural proteins of SARS-CoV-2 have a lower likelihood of inducing humoral responses and neutralizing antibodies, as they are not expressed on the virion surface. However, nonstructural proteins of SARS-CoV-2–infected cells can be presented as MHC-bound peptides and induce cellular immune responses which can be long lasting. Here, using the same workflow, the HLA-A0201–restricted peptide, NSP13-242 (TLVPQEHYV), derived from NSP13 helicase, was identified by MS/MS. Similar to MGP-65, NSP13-242–specific T cells were readily generated using our ETC workflow (Fig. 4*A*). Surprisingly, after stimulation with NSP13-242 peptide, all 48 wells from one 48-well plate showed clear NSP13-242 peptide Tetramer+ CD8+ T cell populations (*SI Appendix*, Fig. S5), suggesting that NSP13-242 peptide may be highly immunogenic.

The cytotoxicity assay also demonstrated that NSP13-242–specific CTLs were able to recognize cognate peptide as low as 100 pM (Fig. 4*B*), indicating expression of high-affinity TCR. More importantly, NSP13-242–specific CTLs were able to lyse NSP13-expressing targets A375-NSP13 and Mel624-NSP13, even at a very low effector to target (E:T) ratio (2.5:1), but not control targets (Fig. 4 *C* and *D*), indicating that NSP13-242–specific CTLs can recognize the endogenously presented peptide of NSP13 protein. Similar to MGP-65 CTLs, the cold target inhibition assay also showed that, when cold targets are added, the lytic capacity of NSP13-242–specific CTLs to hot targets, A375-NSP13 and Mel624-NSP13, was inhibited significantly (Fig. 4 *E* and *F*), further confirming that NSP13-242–specific CTLs lyse the targets via recognition of endogenously presented cognate peptide.

The ICS assay demonstrated that NSP13-242–specific CTLs produce higher levels of inflammatory cytokine IFN-γ and TNF-α and express higher levels of antigen-driven activation markers CD137 and CD69 when cocultured with NSP13-242 peptide-pulsed targets or NSP13-expressing targets, compared with the control targets (Fig. 4*G*). Thus, similar to MGP-65–specific CTLs, NSP13-242–specific CTLs will also initiate a specific cellular immune response when encountering SARS-CoV-2.

### NSP13-448 Peptide-Specific Cytotoxic T Cells Generated from the Peripheral Blood Recognize SARS-CoV-2 NSP13-Expressing Target Cells.

**Fig. 5.**
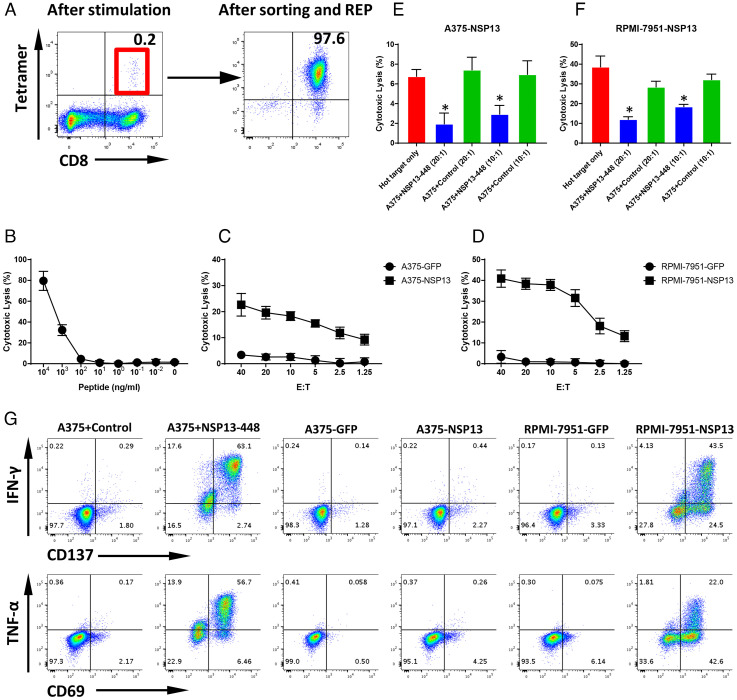
T cell generation and functional validation of nonstructure protein NSP13-derived HLA-A0101 peptide NSP13-448. (*A*) PBMCs from an HLA-A0101 healthy donor were cocultured with NSP13-448 peptide (VYIGDPAQL)–pulsed autologous DCs. After two rounds of stimulation, CD8+ and NSP13-448 Tetramer+ T cells were induced (*Left*). CD8+ and Tetramer+ T cells were sorted and then expanded with REP for 2 wk to generate high-purity NSP13-448–specific CTLs (*Right*). (*B*) The ^51^Cr-labeled A375 cells pulsed with various concentrations of NSP13-448 peptide were cocultured with NSP13-448–specific CTLs at 20:1 E:T ratio. The lysis ability of NSP13-448–specific CTLs was detected with standard CRA. The data are shown as average of triplicate. (*C* and *D*) The ^51^Cr-labeled NSP13 or GFP force expressing HLA-A0101 cell lines A375 (A375-NSP13 and A375-GFP) and RPMI-7951 (RPMI-7951-NSP13 and RPMI-7951-GFP) were cocultured with NSP13-448–specific CTLs at various E:T ratios (from 40:1 to 1.25:1). The lysis ability of NSP13-448–specific CTLs to different targets was detected with standard CRA. The data are shown as average of triplicate. (*E* and *F*) Cold target inhibition assay. The ^51^Cr-labeled A375-NSP13 and RPMI-7951-NSP13 were used as hot targets. Nonradiolabeled A375 cells pulsed with NSP13-448 peptide or irrelevant HLA-A0101 peptide were used as cold targets. The C:H ratio was 10:1 or 20:1. NSP13-448–specific CTLs were cocultured with hot targets alone or hot targets together with cold targets at a 20:1 E:hot T ratio. The lysis ability of NSP13-448–specific CTLs was detected with standard CRA. The data are shown as average of triplicate. (*G*) ICS assay. NSP13-448–specific CTLs were cocultured with A375 pulsed with NSP13-448 peptide or irrelevant HLA-A0101 peptide, as well as A375-NSP13, A375-GFP, RPMI-7951-NSP13, and RPMI-7951-GFP at a 10:1 E:T ratio in the presence of BFA overnight. After incubation, the levels of IFN-γ and TNF-α, as well as TCR pathway downstream activated marker CD137 and CD69, were detected using flow cytometry.

The HLA-A0201 allele is expressed in about 45% of the Caucasian and Asian population (27). Specific T cell targeting of other highly prevalent HLA-A alleles of SARS-CoV-2 would be desirable given the global reach of COVID-19. Using the same workflow for MGP-65 and NSP13-242 peptides, we identified an HLA-A0101–restricted peptide, NSP13-448 (IVDTVSALVY)–derived NSP13 protein, by MS/MS; this allele covers about 26% of Caucasian and 7% of Asian populations (27). Similar to NSP13-242, NSP13-448–specific T cells were readily generated using our ETC workflow (Fig. 5*A*). However, after stimulation with NSP13-448 peptide, only one well from one 48-well plate showed a clear NSP13-448 peptide Tetramer+ CD8+ T cell population (*SI Appendix*, Fig. S6), suggesting that NSP13-448 peptide may not be highly immunogenic.

Cytotoxicity assays also demonstrated that NSP13-448–specific CTLs were able to recognize cognate peptide as low as 100 nM (Fig. 5*B*), indicating moderate to low TCR affinity. Interestingly, NSP13-448–specific CTLs were still able to lyse NSP13-expressing targets A375-NSP13 and RPMI-7951-NSP13, even at a very low E:T ratio (2.5:1), but not control targets (Fig. 5 *C* and *D*), indicating that NSP13-448–specific CTLs can recognize the endogenously presented peptide of NSP13 protein. The cold target inhibition assay demonstrated that, when cold targets are added, the lytic capacity of NSP13-448–specific CTLs to hot targets, A375-NSP13 and RPMI-7951-NSP13, was inhibited significantly (Fig. 5 *E* and *F*), further confirming that NSP13-448–specific CTLs lyse these targets via recognition of endogenously presented cognate peptide.

ICS assays demonstrated that NSP13-448–specific CTLs produce higher levels of inflammatory cytokines, IFN-γ and TNF-α, and express higher levels of antigen-driven activation markers CD137 and CD69 when cocultured with NSP13-448 peptide-pulsed targets or NSP13-expressing targets, compared with control targets (Fig. 5*G*).

### NSP13-134 Peptide-Specific Cytotoxic T Cells Generated from the Peripheral Blood Recognize SARS-CoV-2 NSP13-Expressing Target Cells.

**Fig. 6.**
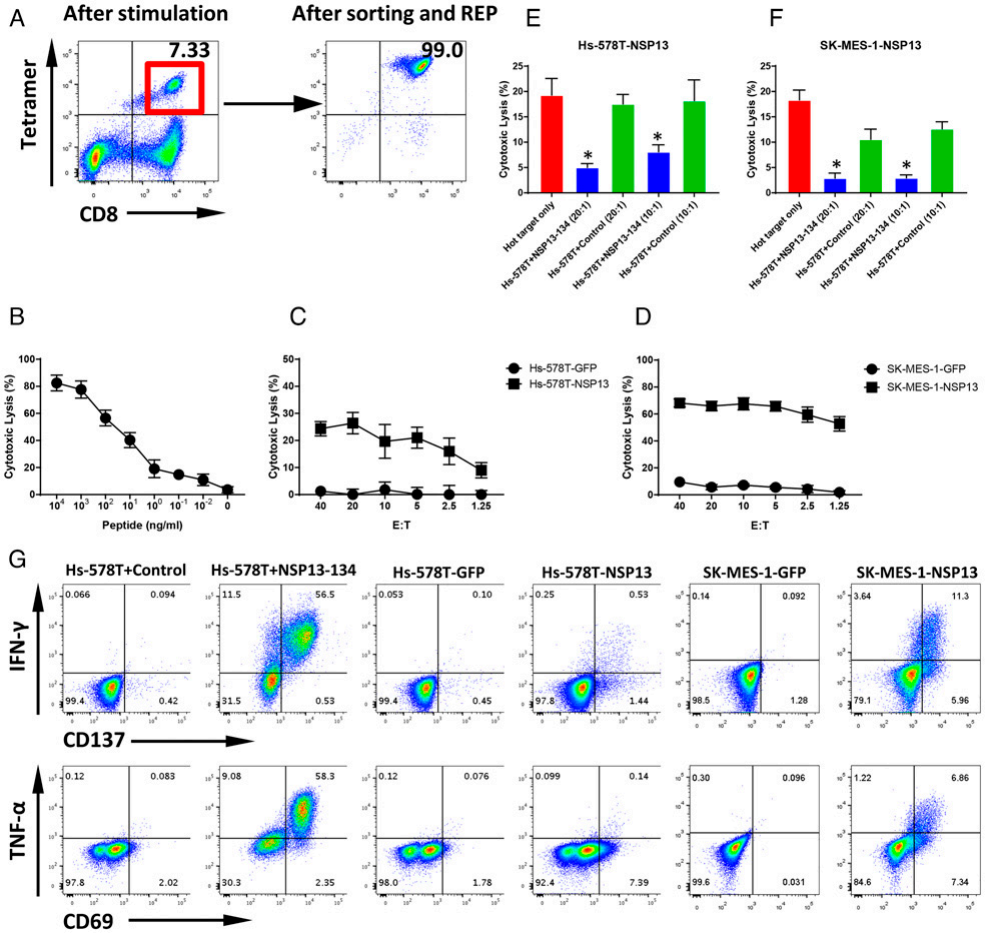
T cell generation and functional validation of nonstructure protein NSP13-derived HLA-A0301 peptide NSP13-134. (*A*) NSP13-134 peptide (KLFAAETLK)–pulsed DCs were cocultured with autologous PBMCs of an HLA-A0301 healthy donor. After two rounds of stimulation, CD8+ and NSP13-134 Tetramer+ T cells were induced (*Left*). CD8+ and Tetramer+ T cells were sorted and then expanded with REP for 2 wk to generate high-purity NSP13-134–specific CTLs (*Right*). (*B*) The ^51^Cr-labeled HLA-A0301 cell lines Hs-578T pulsed with various concentrations of NSP13-134 peptide were cocultured with NSP13-134–specific CTLs at a 20:1 E:T ratio. The lysis ability of NSP13-134–specific CTLs was detected with standard CRA. The data are shown as average of triplicate. (*C* and *D*) The ^51^Cr-labeled NSP13 or GFP force expressing HLA-A0301 cell lines Hs-578T (Hs-578T-NSP13 and Hs-578T-GFP) and SK-MES-1 (SK-MES-1-NSP13 and SK-MES-1-GFP) were cocultured with NSP13-134–specific CTLs at various E:T ratios (from 40:1 to 1.25:1). The lysis ability of NSP13-134–specific CTLs to different targets was detected with standard CRA. The data are shown as average of triplicate. (*E* and *F*) Cold target inhibition assay. The ^51^Cr-labeled Hs-578T-NSP13 and SK-MES-1-NSP13 cells were used as hot targets. Nonradiolabeled Hs-578T cells pulsed with NSP13-134 peptide or irrelevant HLA-A0301 peptide were used as cold targets. The C:H ratio was 10:1 or 20:1. NSP13-134–specific CTLs were cocultured with hot targets alone or hot targets together with cold targets at a 20:1 E:hot T ratio. The lysis ability of NSP13-134–specific CTLs was detected with standard CRA. The data are shown as average of triplicate. (*G*) ICS assay. NSP13-134–specific CTLs were cocultured with Hs-578T pulsed with NSP13-134 peptide or irrelevant HLA-A0301 peptide, as well as Hs-578T-NSP13, Hs-578T-GFP, SK-MES-1-NSP13, and SK-MES-1-GFP at 10:1 E:T ratio in the presence of BFA overnight. After incubation, the levels of IFN-γ and TNF-α, as well as TCR pathway downstream-activated marker CD137 and CD69, were detected using flow cytometry.

In addition to HLA-A0101 and the HLA-A0201 allele, the HLA-A0301 allele covers about 22% of Caucasian and 13% of African populations (27). Using the same workflow, we identified an HLA-A0301–restricted peptide, NSP13-134 (KLFAAETLK)–derived NSP13 protein. Following in vitro stimulation using the ETC workflow, 11 of 48 wells showed a clear NSP13-134 peptide Tetramer+ CD8+ T cell population (*SI Appendix*, Fig. S7), indicating that NSP13-134 peptide is sufficiently immunogenic to induce T cell responses in a healthy donor without prior SARS-CoV-2 infection. After sorting and expansion, high-purity CD8+ and Tetramer+ NSP13-134 CTLs were generated (Fig. 6*A*). The peptide titration assay showed NSP13-134–specific CTLs were able to recognize cognate peptide as low as 100 pM (Fig. 6*B*), indicating expression of relatively high-affinity TCR. Similar to MGP-65 and NSP13-242–specific CTLs, NSP13-134–specific CTLs were able to lyse NSP13-expressing targets Hs-578T-NSP13 and SK-MES-1-NSP13, even at a low E:T ratio (2.5:1), but not control targets (Fig. 6 *C* and *D*). Similar to MGP-65 and NSP13-242 CTLs, the cold target inhibition assay confirmed specific recognition of endogenously presented cognate peptide (Fig. 6 *E* and *F*). The ICS assay also confirmed specific recognition of NS13-expressing targets (Fig. 6*G*).

### Expansion of NSP13-400 Peptide-Specific Cytotoxic T Cells from the Peripheral Blood of Healthy Donors and Functional Assay.

**Fig. 7.**
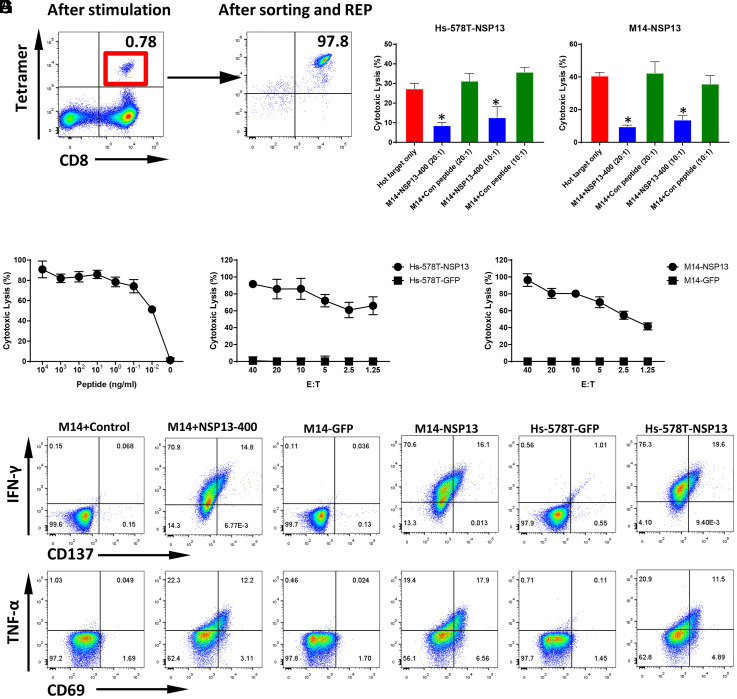
T cell generation and functional validation of nonstructure protein NSP13-derived HLA-A2402 peptide NSP13-400. (*A*) PBMCs from an HLA-A2402 healthy donor were cocultured with NSP13-400 peptide (VYIGDPAQL)–pulsed autologous DCs. After two rounds of stimulation, CD8+ and NSP13-400 Tetramer+ T cells were induced (*Left*). After sorting and REP for CD8+ and Tetramer+ T cells for 2 wk, high-purity NSP13-400–specific CTLs were expanded (*Right*). (*B*) The ^51^Cr-labeled HLA-A2402 M14 cell lines pulsed with various concentrations of NSP13-400 peptide were cocultured with NSP13-400–specific CTLs at a 20:1 E:T ratio. The lysis ability of NSP13-400–specific CTLs was detected with standard CRA. The data are shown as average of triplicate. (*C* and *D*) The ^51^Cr-labeled NSP13 or GFP force expressing HLA-A2402 cell lines Hs-578T (Hs-578T-NSP13 and Hs-578T-GFP) and M14 (M14-NSP13 and M14-GFP) were cocultured with NSP13-400–specific CTLs at various E:T ratios (from 40:1 to 1.25:1). The lysis ability of NSP13-400–specific CTLs to different targets was detected with standard CRA. The data are shown as average of triplicate. (*E* and *F*) Cold target inhibition assay. The ^51^Cr-labeled Hs-578T-NSP13 and M14-NSP13 were used as hot targets. Nonradiolabeled M14 cells pulsed with NSP13-400 peptide or irrelevant HLA-A2402 peptide were used as cold targets. The C:H ratio was 10:1 or 20:1. NSP13-400–specific CTLs were cocultured with hot targets alone or hot targets together with cold targets at a 20:1 E:hot T ratio. The lysis ability of NSP13-242–specific CTLs was detected with standard CRA. The data are shown as average of triplicate. (*G*) ICS assay. NSP13-400–specific CTLs were cocultured with M14 pulsed with NSP13-400 peptide or irrelevant HLA-A2402 peptide, as well as Hs-578T-NSP13, Hs-578T-GFP, M14-NSP13, and M14-GFP at a 10:1 E:T ratio in the presence of BFA overnight. After incubation, the levels of IFN-γ and TNF-α, as well as TCR pathway downstream-activated marker CD137 and CD69, were detected using flow cytometry.

In addition to HLA-A0101, HLA-A0201, and HLA-A0301, the HLA-A2402 allele covers an additional 40% of Asians and 20% of Caucasians (27). Using the same workflow, we identified the HLA-A2402–restricted peptide, NSP13-400 (VYIGDPAQL), of NSP13. Following in vitro stimulation, 9 of 48 wells showed a clear NSP13-400 peptide Tetramer+ CD8+ T cell population (*SI Appendix*, Fig. S8). After sorting and expansion, high-purity CD8+ and Tetramer+ NSP13-400 CTLs were generated (Fig. 7*A*). Similar to MGP-65 CTLs, NSP13-400–specific CTLs showed very high recognition affinity for the cognate peptide in the peptide titration assay, as low as 10-pM concentration (Fig. 7*B*), and, in accordance with the peptide titration assay, very high, specific lysis of NSP13-expressing targets Hs-578T-NSP13 and M14-NSP13, even at E:T ratios as low as 1.25:1 (Fig. 7 *C* and *D*). Similarly, the cold target inhibition assay showed specific levels of NSP13-400 CTLs to hot target Hs-578T-NSP13 and M14-NSP13 which were significantly inhibited with the addition of cold targets (Fig. 7 *E* and *F*), further confirming that NSP13-400–specific CTLs lyse the SARS-CoV-2 targets via recognition of endogenously presented cognate peptide.

The ICS assay also confirmed specific recognition of NS13-expressing targets (Fig. 7*G*). IFN-γ and TNF-α levels were strikingly elevated in comparison with other SARS-CoV-2 CTL target assays, suggesting high-density endogenous presentation.

In summary, using our MHC immunoprecipitation elution and MS identification workflow, we discovered five HLA class I–restricted peptides derived from structure protein MGP and nonstructure protein NSP13 of SARS-CoV-2 presented by several HLA alleles (HLA-A0101, HLA-A0201, HLA-A0301, and HLA-A2402) which cover ∼80% of Caucasian and Asian populations. All five peptides were highly immunogenic and capable of readily eliciting T cell responses among healthy COVID-19–negative donors. All five SARS-CoV-2–specific CTLs recognize endogenously presented cognate peptide and specifically lyse SARS-CoV-2 + targets.

### MGP-65–Specific TCR-Ts Recognize SARS-CoV-2 MGP-65–Expressing Target Cells.

**Fig. 8.**
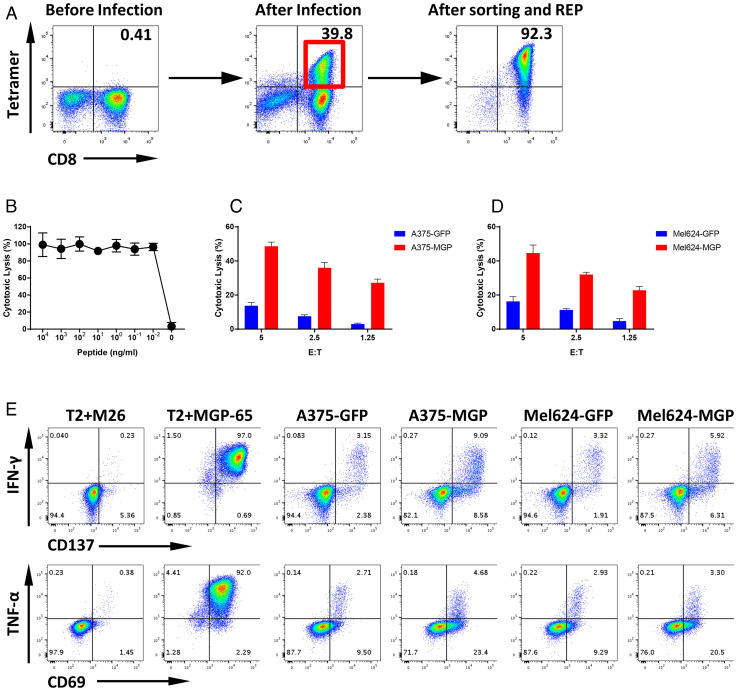
MGP-65 peptide-specific TCR-T generation and functional validation. (*A*) The whole length of the MGP-65 TCR alpha chain and beta chain linked with FP2A peptide was inserted into retrovirus vector pMSGV1, and then the recombinant retrovirus vector was used to infect the OKT3 activated HLA-A0201 allo-PBMCs (*Left*). After about 5 d of infection, CD8+ and MGP-65-Tetramer+ T cell populations were analyzed by flow cytometry (*Middle*). CD8+ and MGP-65 Tetramer+ cells were then sorted and expanded with REP. After expansion for 2 wk, high-purity CTLs (Tetramer+ population over 90%) were generated (*Right*). (*B*) Peptide titration assay for MGP-65–specific TCR-T. The ^51^Cr-labeled T2 cells pulsed with various concentrations of MGP-65 peptide were cocultured with MGP-65–specific TCR-T. The lysis level of MGP-65–specific TCR-T was analyzed with standard CRA. The data are shown as average of triplicate. (*C* and *D*) Antigen-specific cytolysis analysis for MGP-65–specific TCR-T. The ^51^Cr-labeled A375-MGP, A375-GFP, Mel624-MGP, or Mel624-GFP was cocultured with MGP-65–specific TCR-T at various E:T ratios. The lysis level of MGP-65–specific TCR-T to different targets was analyzed with standard CRA. The data are shown as average of triplicate. (*E*) ICS assay. MGP-65–specific TCR-Ts were cocultured with T2 pulsed with MGP-65 peptide or M26 irrelevant peptide, as well as A375-MGP, A375-GFP, Mel624-MGP, and Mel624-GFP at a 10:1 E:T ratio in the presence of BFA overnight. After incubation, the levels of IFN-γ and TNF-α, as well as TCR pathway downstream activated marker CD137 and CD69, were detected using flow cytometry.

As proof of principle that these discoveries can lead to development of “off-the-shelf” SARS-CoV-2–specific T cell therapy of COVID-19 patients, we sequenced and cloned the TCR alpha and beta chains from MGP-65–specific T cells and determined whether it was possible to transfer specificity and function to peripheral blood lymphocytes (PBLs). The sequence annotation revealed an alpha chain (TCR-α) belonging to the TRAV17*01F/TRAJ50*01F subtype and a beta chain (TCR-β) belonging to TRBV9*02F/TRBJ2-1*01F/TRBD1*01F(*SI Appendix*, Fig. S9). The retroviral vector pMSGV1 containing the whole length of the TCR alpha chain and beta chain linked with cleavage peptide Furin and P2A was constructed and used to infect OKT3 activated allogeneic PBLs of another HLA-A0201 healthy donor. After 5 d of infection, about 37% CD8+Tetramer+ T cell population was observed (Fig. 8*A*), indicating successful exogenous TCR pairing in allogeneic PBLs. After sorting and expansion, high-purity MGP-65–specific TCR-Ts were generated (Fig. 8*A*).

To evaluate the function and specificity of MGP-65–specific TCR-Ts, a cytotoxicity CRA and an ICS assay were performed and compared to the parental MGP-65–specific CTL line. MGP-65 TCR-Ts were able to recognize titrated peptide-pulsing targets at peptide concentrations as low as 10 pM (Fig. 8*B*), indicating that the TCR-Ts also displayed high-affinity recognition of cognate peptide. Furthermore, MGP-65 TCR-Ts specifically lysed MGP-expressing targets A375-MGP and M624-MGP (Fig. 8 *C* and *D*), commensurate with ICS assays, demonstrating that, compared to the parental CTLs, MGP-65–specific TCR-Ts produced higher levels of IFN-γ and TNF-α, as well as activation markers CD137 and CD69, when cocultured with MGP-expressing targets or MGP-65 peptide-pulsing targets (Fig. 8*E*).

## Discussion

To date, nearly 1,500 predicted class I epitopes for SARS-CoV-2 have been identified by in silico prediction methods, and, in some cases, “validated” by eliciting T cell responses using PBMCs of patients with COVID-19 (4, 5). These peptides have been used extensively to evaluate the T cell response of patients, and occasionally healthy donors, to COVID-19, and COVID-19 vaccines, and, increasingly, to develop T cell–based therapies. What has not been demonstrated, however, is whether any of these 1,500 predicted peptides are, in fact, processed and presented by SARS-CoV-2+ cells and represent naturally occurring epitopes recognized by T cells. A preliminary screen of predicted SARS-CoV-2 epitopes considered “immunodominant” among widely cited reports appears to support this premise: We found that seven of these eight predicted peptides were unable to elicit a T cell response that would lead to recognition of SARS-CoV-2+ targets, suggesting that responses to these peptides may be artifactual or, at best, cross-reactive (Fig. 1) (4, 7, 15–22). To date, there has been no empiric validation of SARS-CoV-2 epitopes for immunogenicity.

We postulate that immunogenic epitopes for SARS-CoV-2 are best defined empirically by directly analyzing peptides eluted from MHC and then validating immunogenicity by determining whether such peptides can elicit T cells recognizing SARS-CoV-2 antigen-expressing targets. MS is an ideal analytical approach to precisely identify the naturally expressed antigenic epitopes and enables investigators to address the complexity associated with differential expression and processing of antigenic proteins by infected cells. Based on immunoaffinity capture of the MHC-antigenic peptide complex from cells engineered to express SARS-CoV-2 genes, our approach allows for direct profiling and identification of the SARS-CoV-2 immunopeptidome. By eliciting T cell responses against these candidate epitopes, we confirm empiric recognition of SARS-CoV-2+ cells and endogenous presentation of these peptides.

In this study, we identify and validate five class I–restricted SARS-CoV-2 epitopes expressed by structural (MGP) and nonstructural (NSP13) genes, presented by class I alleles (HLA-A*0101, A*0201, A*0301, and HLA-A*2402) prevalent among >75% of the general population. Using recombinant vectors encoding these alleles, we engineered the expression of highly conserved regions of SARS-CoV-2 MGP and nonstructural protein-13 (NSP13) genes, recovered MHC, eluted peptides, and applied DDA MS/MS, yielding over 12,000 spectra, which were then deconvoluted and filtered to a handful of candidate peptide epitopes. The immunogenicity of five peptides was validated on the basis of their ability to elicit peptide-specific T cells capable of recognizing and killing SARS-CoV-2–expressing target cells, and, in one example, redirecting specificity of PBLs with an engineered TCR to SARS-CoV-2 MGP.

The importance of eliciting a meaningful antiviral T cell response has been well documented; SARS- and MERS-responsive T cells were found to have a protective role (28). Emergence of SARS-CoV-2–specific T cell responses was recently shown to be associated with a sustained viral clearance and highlight the importance of developing vaccines that promote cellular immunity against SARS-CoV-2 (29–31).

Recently, the emergence of mutant escape variants of SARS-CoV-2 has led to global concerns over possible breaches in viral protection following immunization with current vaccines which elicits a predominantly serologic response(15, 32–35). By targeting a nonsurface, nonstructural protein, in this case, nsp13, which encodes viral helicase, escape variants are less likely to develop; in fact, none of the known variants harbor mutations among the epitope sequences identified here. Furthermore, the strategy presented allows for identification of epitopes spanning almost any SARS-CoV-2 gene; selection of virus-essential gene targets provides a rational T cell–based approach to mitigate selection of an antigen-loss variant and the potential for long-term viral immunoprotection.

Equally important in defining the landscape of COVID-19 infection and control, its natural history, vaccine efficacy, and therapeutic intervention, is an accurate measure of the SARS-CoV-2–specific immune response. While the pools of predicted peptides currently in use to evaluate class II– and class I–restricted responses have been used extensively and appear to provide a measure of overall immune response, SARS-CoV-2–specific immunity is poorly defined when the majority of peptides may not be immunogenic; the use of a highly defined subset of peptides may provide a more accurate representation of T cell immune response to COVID-19 infection. Although our current panel of five peptides is not extensive, it does represent highly conserved regions of the SARS-CoV-2 genome, presented by several highly prevalent allelotypes, and may readily be applied to class II– as well as class I–restricted epitopes. A more extensive panel of 18 epitopes has been prepared and will be evaluated for clinical correlative studies.

Finally, as further proof of immunogenicity for epitopes defined in this manner (by tandem MS followed by empiric in vitro validation), we reconstituted functional SARS-CoV-2–specific TCRs using a vector encoding the alpha and beta chains of MGP-65–specific T cells. This strategy additionally provides an off-the-shelf reagent for adoptive TCR-T–based therapies, and one can envision a collection of TCR-T vectors recognizing a matrix of MS/MS-defined SARS-CoV-2 epitopes spanning highly conserved regions of the viral genome and representing a broad panel of high-prevalence HLA alleles for cell-based therapy of COVID-19–infected patients.

One limitation of this study is that we only identified HLA class I epitopes but not HLA class II epitopes recognized by CD4+ T cells which are equally important for adaptive immunity. Antigen-specific CD4+ T cells not only prime B cells and launch humoral immune responses against SARS-CoV-2 but also provide helper function to CD8 T cells in viral infections and cancer. Several studies have demonstrated the important role of CD4+ T cell response for COVID-19 patients (6, 10–13). A similar strategy can be applied to elute class II–restricted peptides for mass spectrometric analysis and epitope validation.

Furthermore, additional epitopes to these and other nonstructural regions would be desirable and broaden the panel for analysis of T cell–based responses as well as downstream development of vaccine and T cell therapeutics. Because of lacking a BSL3 facility, the T cell functional assays were performed using pseudotyped virus-infected cell targets (rather than live SARS-CoV-2 virus infection), which, while suitable for antigen presentation, does not preclude the prospect of other mechanisms of immune escape in cells infected with live virus.

## Materials and Methods

For the cell lines, reagents, peptide identification, T cell generation, and functional validation, please see *SI Appendix*.

## Data Availability

All study data are included in the article and *SI Appendix*.
